# Two cases of refractory *Cryptococcus gatti* (*C gatti*) infection and literature review: Case report

**DOI:** 10.1097/MD.0000000000046999

**Published:** 2026-01-30

**Authors:** Yanting Zeng, Tiegen Xiong, Wei Xiang, Jiake Zhang, Li Li, Weifeng Yuan, Wenyuan He, Bingmei Deng, Hong Xu

**Affiliations:** a Department of Infectious Medicine, General Hospital of Southern Theater Command of PLA, Guangzhou, China; b Department of Neurology, General Hospital of Southern Theater Command of PLA, Guangzhou, China; c Department of Respiratory Medicine, The First Affiliated Hospital of Guangdong Pharmaceutical University, Guangzhou, Guangdong, China; d Department of Pathology, General Hospital of Southern Theater Command of PLA, Guangzhou, China; e Department of Pulmonary and Critical Care Medicine, General Hospital of Southern Theater Command of PLA, Guangzhou, China.

**Keywords:** *C gatti*, case report, encephalitis, pulmonary cryptococcosis, transbronchial lung biopsy

## Abstract

**Rationale:**

The incidence of *Cryptococcus gatti* (*C gatti*) infection has been increasing, posing significant challenges in clinical diagnosis and treatment due to its high virulence, refractoriness, and mortality.

**Patient concerns:**

Both patients had no immune function defects. One of the cryptococcal drug sensitivity results suggested resistance to fluconazole, which ultimately required surgical intervention. Two patients had recurrent symptoms and worsened examination results during antifungal treatment, and the course of anti-infection was more than 6 months.

**Diagnoses:**

Pulmonary cryptococcosis complicated with cryptococcal encephalitis caused by *C gatti*.

**Interventions:**

We used standard treatment in induction, consolidation, and maintenance periods, dynamically evaluating efficacy, monitoring fluconazole MIC, and timely surgical intervention.

**Outcomes:**

Two patients recovered after treatment.

**Lessons:**

A literature review was conducted using PubMed, China National Knowledge Infrastructure, Wanfang, and VIP databases with the search term “gatti” to identify reported cases both domestically and internationally. Relevant data were extracted for further analysis. The treatment of *C gatti* is challenging. We need to continuously adjust the treatment plan based on the patient’s symptoms, test results, and response to treatment to obtain an accurate diagnosis and maximize treatment effectiveness.

## 1. Introduction

We report 2 cases of pulmonary cryptococcosis complicated with cryptococcal encephalitis caused by *C gatti*. The first female patient still had new neurological symptoms and increased lung lesions after 2 months of standard induction therapy with antifungal agents. Cryptococcus smear indicates that the number of Cryptococcus is gradually increasing. The second male patient experienced an increase in intracranial lesions after 2 months of treatment, with no significant absorption within the pulmonary lesions. Surgical intervention was required to remove the pulmonary lesions. Both patients were infected with *C gatti* in the lungs and brain, but neither exhibited signs of immune compromise. They both required prolonged hospitalization for diagnosis and treatment, with suboptimal outcomes. These why we call them “refractory.”

## 2. Case 1

The patient was a 48-year-old female. She was admitted to the hospital due to fever, headache, dizziness and vomiting for 20 days. The patient had free-range chickens (as pets) at her house, and occasionally pigeons fly in. These animals might have been potential sources of infection. On September 15, 2023, she developed persistent forehead pain and dizziness, accompanied by vomiting in severe cases. Her body temperature fluctuated between 37°C and 37.5°C. During physical examination, wet rales were heard in the left upper lung. She was conscious, with normal memory and calculation ability, and cooperated well with the physical examination. Her speech was clear, and she answered questions to the point. The pupils of both eyes were equal in size and round, with a diameter of about 3 mm. They had sensitive light reflexes, normal eye movements, symmetrical bilateral forehead lines and nasolabial grooves, normal pharyngeal reflexes, and the tongue was centered when extended. Neck stiffness and a positive Kernig sign were noted. The muscle tension of the limbs was normal, with a muscle strength level of 5. Bilateral tendon reflexes were normal, and the Babinski sign was negative. The Finger nose test, heel knee tibia test, and rotation test were normal, and she had no difficulty standing upright with eyes closed.

On September 28, lumbar puncture revealed a cerebrospinal fluid (CSF) pressure of 225 mmH_2_O (80–180 mmH_2_O). The CSF examination showed “white blood cells 863 × 10^6^/L, CSF protein 1540.3 mg/L, and weak positive Rivalta test.” Despite symptomatic treatment (specific details unknown), her symptoms did not improve. On October 4, she underwent another lumbar puncture at Zhongshan People’s Hospital. The CSF metagenomic next generation sequencing (mNGS) test indicated the presence of *Cryptococcus gatti* (*C gatti*) VG I type with a sequence reads count of 235. The initial diagnosis was “Cryptococcal meningitis.” After receiving fluconazole antifungal treatment, her symptoms improved.

**Figure 1.**
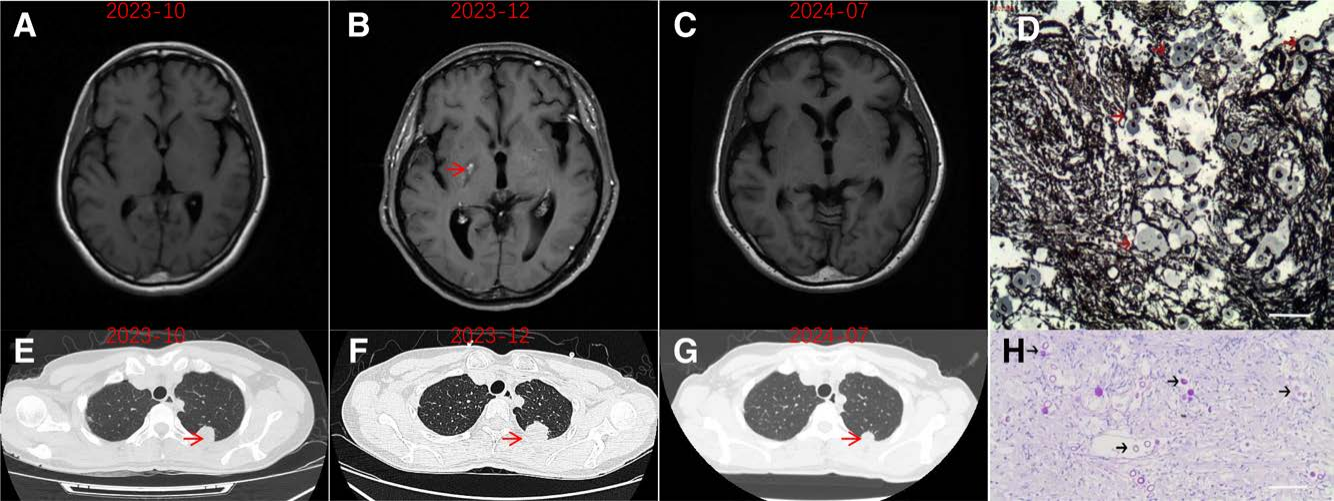
(A, B, C) MRI images of case 1; (A and C) normal. (B) Multiple abnormal signals in the brain and pia mater, newly detected compared to image (A). (E, F, G) CT images of case 1, which show left upper lung nodule. (D) Lung biopsy tissue, and the fungus in the alveoli are positive by Grocott methenamine silver (GMS) (red arrow). (H) Lung biopsy tissue, and the fungus in the alveoli are positive by PAS staining (black arrow). Bar in (D) and (H) = 10 μm. CT = computer tomography, MRI = magnetic resonance imaging, PAS = periodic acid-schiff.

For further diagnosis and treatment, she was admitted to our hospital’s neurology department on October 6 (Fig. 1A and E). After admission, the patient was treated with amphotericin B (30 mg/d) + flucytosine (5-FC) (1.5 g tid) and lumbar puncture was performed the next day. Compared to when she first suffered from the disease, the patient’s CSF pressure decreased but remained high. Her symptoms of headache, nausea and vomiting improved. Since admission, CSF white blood cells, sugar, and chloride showed improvement.

However, on December 5, she experienced repeated episodes of left lower limb weakness during activities, with a total of 6 episodes, each lasting about ten minutes. That’s what confused us. Re-examination of cranial magnetic resonance imaging (MRI) and chest computer tomography (CT) revealed new lesions (Fig. 1B and F). CSF smear showed a gradual increase in the number of Cryptococcus, but all cultures were negative. After 2 months of antifungal treatment with a total dose of 1.88 g amphotericin B, the patient’s CSF Cryptococcus increased, and clinical symptoms partially alleviated, but some new symptoms appeared. December 11, 2023, fluconazole (0.4 g qd) was added for triple anticryptococcus treatment. On December 26, 2023, CT-guided lung biopsy was performed. Pathology showed interstitial fibrous tissue and fibroblast proliferation with chronic inflammatory cell infiltration, sporadic spore-like material in the alveolar cavity, periodic acid-schiff (+), periodic acid-silver methenamine (+), acid fast (−), Gram stain (−) (Fig. 1D and H), which was consistent with cryptococcal infection. On December 25, 2023, the total dose of amphotericin B reached 2 g, and anticryptococcus treatment was adjusted to fluconazole 800 mg/d + flucytosine (1.5 g tid). The patient’s CSF white blood cells, sugar, and chloride further improved.

**Figure 2.**
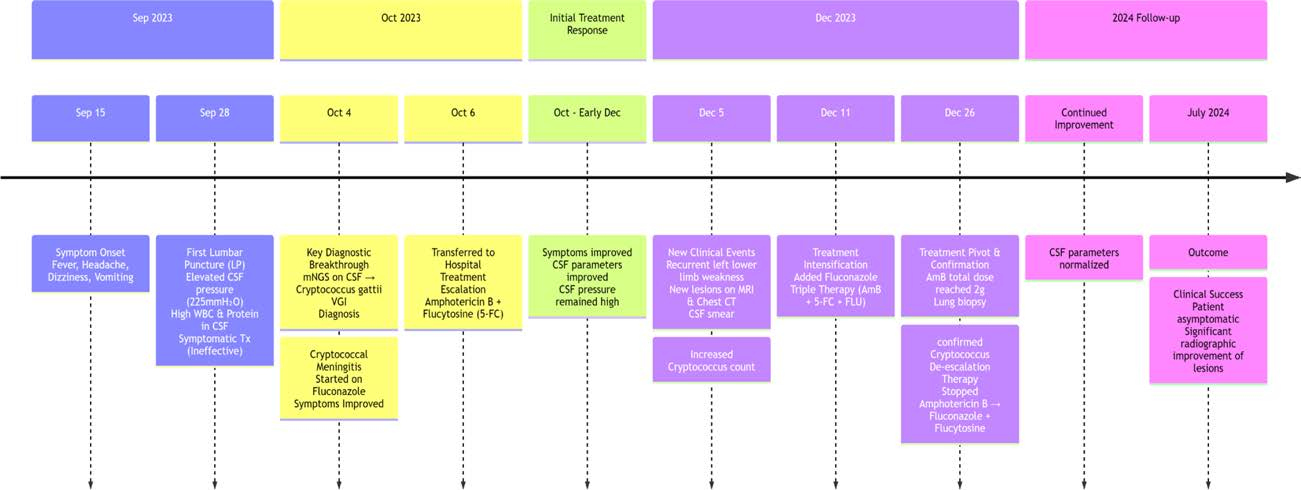
Case 1 clinical timeline.

Outcome: The patient was followed up on July 2024 (Fig. 1C and G) and complained of no discomfort. Re-examination showed significant improvement in the pulmonary and intracranial lesions. In February 2025, oral antifungal agents was discontinued. The total treatment course was 14 months (Figs. 1 and 2).

## 3. Case 2

**Figure 3.**
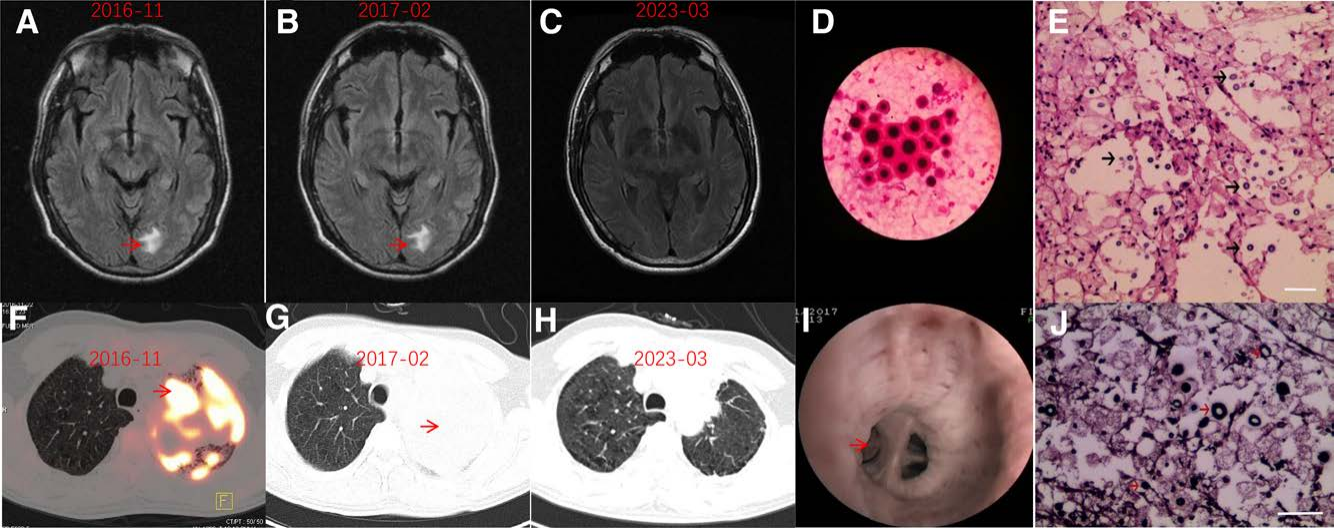
(A, B, C) MRI images of case 2, which suggest left occipital lobe lesion. (F) PET-CT image of case 2, showing hypermetabolic lesion in upper left lung. (G) CT image of case 2 showing no significant improvement in the left upper lung lesion after antifungal treatment. (H) The lesion in the upper left lung has been surgically removed. (D) Cryptococcus was found in alveolar lavage. (I) Polypoid changes of the upper lobe airway of the left lung revealed by fibrobronchoscopy. (E) Hematoxylin–eosin staining of lung biopsy tissue showing infiltration of neutrophils and lymphocytes in lung tissue, proliferation of interstitial fibrous tissue, infiltration and aggregation of foam like histiocytes in alveoli, and scattered round or oval fungus (black arrows) in alveoli. The fungus had thick capsules, and the center was empty and bright. (J) The fungus in the alveoli were positive by GMS staining, and were surrounded by thick mucoid (red arrow). Bar in (E) and (J) = 10 μm. CT = computer tomography, GMS = Grocott methenamine silver, MRI = magnetic resonance imaging, PET-CT = Positron emission tomography–computer tomography.

This patient was a 49-year-old male who was engaged in aluminum smelting work and had a history of exposure to small amounts of chemical fumes. He had contact with dead mice 3 months before the onset of the disease and also had a history of smoking. Initially, in October 2016, the patient experienced left chest pain, which was mild and paroxysmal. He could tolerate the pain without medical treatment, but the symptoms recurred repeatedly. One month before admission, the patient developed a low fever. The chest pain remained unchanged, and there were no symptoms such as joint pain or chest tightness. On November 5, due to unrelieved chest pain, he was admitted to another hospital for examination and was found to have left upper pneumonia. He was given anti-infective therapy (the specific medication was not provided at the time). After about 1 week of treatment, his body temperature returned to normal, but the chest pain persisted and its nature was similar to that before. Positron emission tomography–computer tomography (PET-CT) indicated the possibility of lung malignancy (Fig. 3A and F). A lung biopsy was performed, and pathological considerations suggested possible cryptococcal infection. Fluconazole (400 mg/d) was administered for further treatment.

Subsequently, on December 2, he was transferred to our hospital for further treatment due to fear of developing a malignant lung tumor. His chest CT showed a solid shadow in the left upper lung, with an increased white blood cell count and elevated erythrocyte sedimentation rate, indicating a possible infection. However, the procalcitonin (PCT) concentration was normal, which suggested that the infection might not be bacterial in nature. Human Immunodeficiency Virus (HIV) test was negative. Follow-up blood tests revealed weak positive results for cryptococcal antigen. The chest CT scan showed that the lesion in the left upper lung had slightly shrunk, and the patient was discharged on oral fluconazole (400 mg/d) therapy. Later, on January 1, 2017, the patient had fever and headache again with a temperature of about 38°C. The fever was more pronounced in the afternoon while normal in the morning. Oral administration of ibuprofen could reduce the fever quickly, but it recurred. On January 3, he was admitted to the hospital again. The left upper lung lesion had slightly enlarged. On January 4, 2017, fibrobronchoscopy showed polypoid changes in the upper lobe airway of the left lung (Fig. 3I), and Cryptococcus was found in alveolar lavage (Fig. 3D).

Drug susceptibility test (CLSI criteria) showed that Cryptococcus was resistant to fluconazole and itraconazole, intermediate to flucytosine, and sensitive to amphotericin B and voriconazole. Our hospital's mass spectrometry (BioMerieux, Marcy l'Etoile, France) identified the strain as Cryptococcus neoformans (database V2.0) with a confidence level of 96%. The blood Cryptococcus antigen titer was 1:1280, and cranial MRI indicated a fungal brain abscess, although metastatic tumors could not be ruled out. His blood test showed an elevated Cryptococcus antigen titer, and the CSF pressure was 170 mmH_2_O. The CSF Cryptococcus antigen test was positive. Based on the drug susceptibility results, treatment with amphotericin B (30 mg/d) + flucytosine (5-FC) (1.5 g tid) was initiated. Posttreatment chest CT scans showed a reduction in the size of the lesion. However, the patient experienced an increase in blood creatinine concentration, likely an adverse effect of amphotericin B, and developed a new headache. Voriconazole antifungal therapy was started on January 17, 2017. Despite this, chest CT scans showed no significant reduction in the left upper lung lesion.

Given the limited efficacy of antifungal treatments and the potential risk to the patient’s life if Cryptococcus resistance progressed, a hospital-wide consultation was held. Excluding absolute contraindications to surgery, a thoracoscopic-assisted left upper lobectomy was performed under general anesthesia on February 13, 2017 (Fig. 3B and G).

The patient recovered well postoperatively. Postoperative pathology confirmed that the left upper lung mass was consistent with cryptococcosis. Immunohistochemistry results were CK(+) in epithelial cells and CD68(+) in histiocytes. Special staining results were periodic acid-schiff (+) and periodic acid-silver methenamine (+) (Fig. 3E and J).

The white blood cell count in the routine blood test returned to normal, and the Cryptococcus antigen titer decreased to 1:640 by March 8, 2017. On March 9, 2017, a cranial MRI showed a reduction in brain lesions, and the patient was subsequently discharged. A reexamination on April 6, 2017, revealed no recurrence of infection in the lungs via chest CT, but cranial MRI showed similar lesions compared to previous findings, and the Cryptococcus antigen titer remained at 1:640. Oral voriconazole was discontinued after 2 months.

Despite the initial identification of the strain as Cryptococcus neoformans, the clinical treatment response did not match expectations, as previous diagnoses of Cryptococcus neoformans had shown good therapeutic outcomes. This discrepancy led to skepticism regarding the identification results. In 2020, our hospital’s mass spectrometer (MS) database (VITEKMS) was updated from V2.0 to V3.0. Upon reevaluation, it was discovered that the database update had introduced biases in many previous pathogen data. The laboratory director re-cultured the patient’s strain and performed mass spectrometry identification, which confirmed it as *C gatti* with a confidence level of 99.9%.

**Figure 4.**
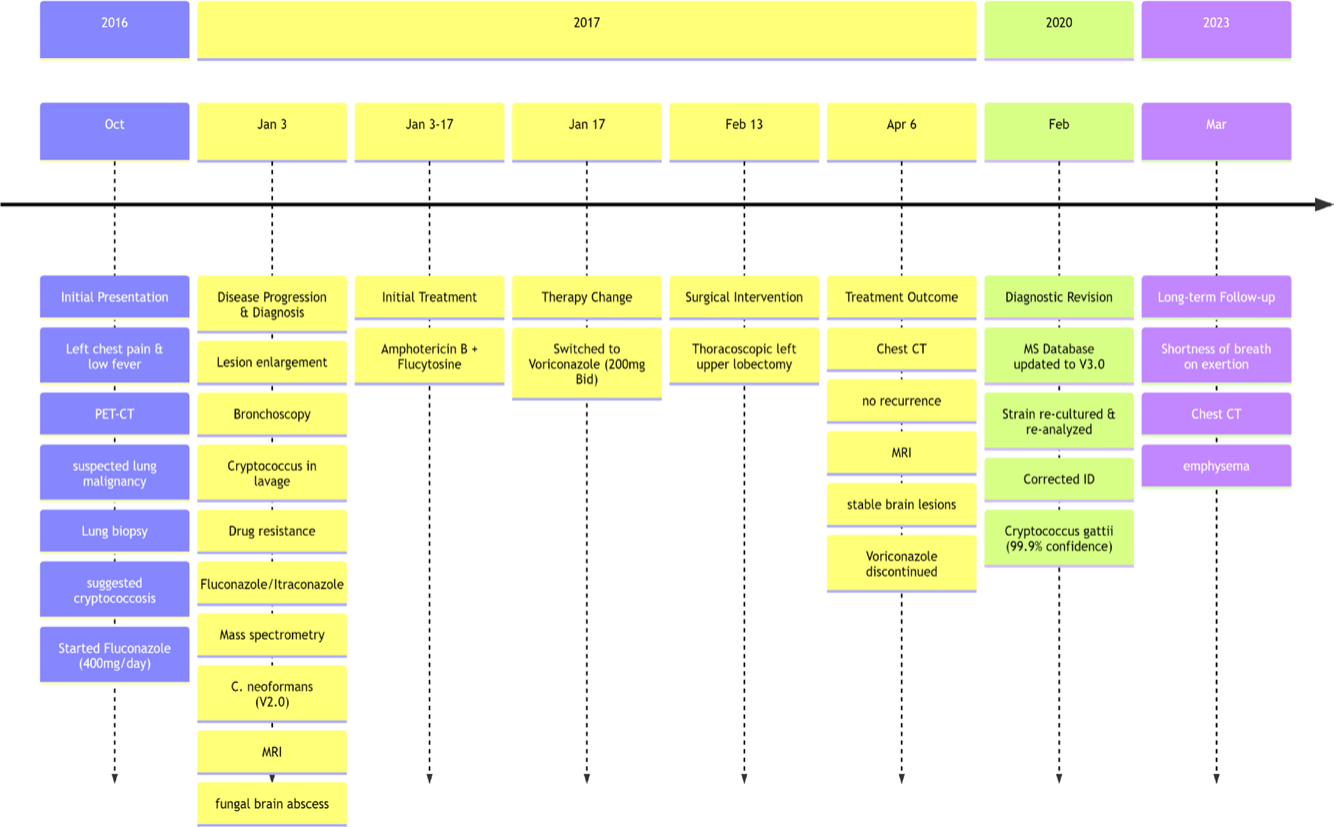
Case 2 clinical timeline.

Outcome: The total course of antifungal treatment was 8 months. During a follow-up visit in 2023, the patient reported shortness of breath after physical activity. Chest CT scan revealed emphysema, and brain MRI shown that compared to the previous image, the abnormal signal in the right occipital lobe has disappeared (Fig. 3C and H). Pulmonary function test indicated severe mixed pulmonary ventilation dysfunction, predominantly obstructive. The patient was prescribed long-term inhalation of bronchodilators. Follow-up visit in 2024, the patient reported slight improvement in shortness of breath, no fever or headache (Figs. 3 and 4).

## 4. Literature review

The search term “gatti” was used to retrieve articles from the PubMed, China National Knowledge Infrastructure, Wanfang, and VIP databases. The time range of retrieval spans from the earliest available records in the databases until February 1, 2024. We first read all the literature abstracts obtained from the search and screened for articles reporting confirmed or suspected *C gatti* infections. We then checked the reference lists of these articles to expand our literature sources and conducted further screening. After identifying the target literature, we read the full texts, extracted relevant data, and merged data from repeated cases. After a thorough review and screening process, we included 14 articles, which reported a total of 14 cases, including 6 Chinese and 8 foreign cases.^[1–14]^ The case data are shown in Table 1.

**Table 1:** Clinical data of 14 patients with *C gattii* infection.

Ref	Age	Sex	Clinical manifestation	Diagnostic method	Treatment	Outcome
^[1]^	49	F	Discovered pulmonary nodules	Operation	Operation	Survived
^[2]^	29	M	Dizziness, gibberish	CSF culture	L-AmB + 5-FC + GC for 6 wk	Survived
^[3]^	62	M	Headache, fever, nodules in lungs	CGB agar culture, Vitek 2, MALDI-TOF MS, ITS and IGS Sequence analysis	AmB + FLU + 5-FC, for 4 wk; FLU for 1 yr. Intrathecal injection of AmB.	Survived
^[4]^	33	M	Fever, headache, ghosting, nodules in lungs	CSF mNGS	AmB + FLU for 4 wk; FLU + 5-FC for 1 yr, VPS, Intrathecal injection of Am B + GC	Survived
^[5]^	46	F	Fever, headache, nodules in lungs	CSF mNGS:VGIII	L-AmB + FLU	Survived
^[6]^	21	M	Fever, headache, consciousness disorders	18S rRNA sequence, phylogenetic tree analysis	AmB + FLU + 5-FC	Survived
^[7]^	52	M	Headache, nausea, vomiting, vision changes.	CSF cryptococcus antigen (+), CSF PCR for *Cryptococcus neoformans*(−)	L-AmB + 5-FC for 4 wk, FLU for 1 yr. Keppra for seizure prophylaxis	Survived
^[8]^	23	F	Headache, nausea, tinnitus, and blurred vision.	CSF were positive for *C gatti*.	AmB + 5-FC for 4 wk, FLU for 1 yr, lumbar drain	Survived
^[9]^	80	M	Lesion on right forearm	skin smears, *C gatti*, serotype B	FLU for 20 wk	Survived
^[10]^	53	F	Flaccid paralysis, peripheral nerve involvement	CSF molecular analysis	AmB-D + FLU for 2 wk. FLU for 6 wk	Survived
^[11]^	64	M	Cryptococcal sinusitis	PET-CT , histopathology	Surgical excision. Bortezomib + lenalidomide + GC	Survived
^[12]^	27	M	Headache, weight loss	CGB agar culture, biochemical reaction and serotyping.	AmB + FLU. Antiretroviral treatment	Dead at day 8
^[13]^	13	M	Fever, breathlessness, headache, ataxic gait	Vitek 2, 18S rRNA sequencing technique	AmB + FLU, mechanical ventilation	Dead at day 8.
^[14]^	24	F	Multifocal encephalomyelitis, nodules in lungs	biopsy of brain	AmB + 5-FC + GC for 4 wk, FLU for 2 yr	Survived

5-FC = flucytosine, AmB = amphotericin B, CGB = canavanine-glycine-bromothymolblue, CSF = cerebrospinal fluid, F = female, FLU = fluconazole, GC = glucocorticoids, L-AmB = Liposomal Amphotericin B, M = male, MALDI-TOF MS = matrix-assisted laser desorption/ionization time-of-flight mass spectrometry, PET-CT = Positron emission tomography–computer tomography, VPS = ventriculoperitoneal shunt.

Among the 14 patients, there were 9 males and 5 females, with ages ranging from 13 to 80 years old. The median age was 39.5 years. The cases included 8 instances of intracranial infection, 1 case of pulmonary infection, 3 cases of concurrent lung and intracranial infections, 1 case of sinusitis, and 1 case of skin infection. There were also 2 deaths, both of which were related to the progression of *C gatti* infection. It is worth noting that only 2 cases reported the drug susceptibility results of *C gatti* in the literature. Both strains were sensitive to commonly used antifungal agents, as determined by standard disk diffusion assays. This limited reporting may be due to the lack of standardized laboratory facilities or reporting practices in some studies.

## 5. Discussion

*C gatti* was previously considered to be a variant of Cryptococcus neoformans, but it was identified as an independent species in 2002. *C gatti* is inhaled through basidiospores and yeast cells. There are 4 molecular types. Types I and II are associated with healthy hosts, while types III and IV are linked to HIV in Africa. The 2 species of Cryptococcus are closely related but also different. Cryptococcus neoformans is distributed worldwide, while *C gatti* is mainly found in North America and Australia. The incidence rate of *C gatti* infections ranges from 1 to 11%. It has more neurological complications, and the efficacy of antifungal treatment is worse than that of Cryptococcus neoformans.^[15]^ Cryptococcus neoformans is mainly detected from bird feces, with a total detection rate of 12.9%. *C gatti* is mainly isolated from trees and rotten wood, and has been found in Jiangxi, Guangdong, Guangxi, Zhejiang, and other places in China. Clinical characteristics of *C gatti* include: more likely to occur in immunocompetent hosts, more susceptible to encephalitis and meningitis, more prone to form cryptococcosis tumors (in the brain and lungs), imaging similar to that of Cryptococcus neoformans, more susceptible to drug resistance, and prolonged anti-infection time.

Therapeutic challenges: Poor therapeutic outcomes in patients may be attributed to drug resistance or the dormant state of *C gatti*. Among the currently effective drugs for Cryptococcus, polyenes directly target ergosterol, pyrimidine analogs block DNA and RNA synthesis, and azoles such as fluconazole disrupt membrane integrity by inhibiting the ergosterol biosynthesis enzyme Erg11.^[16]^ The mechanism of resistance to fluconazole in *C gatti* is unclear, but it may be related to overexpression of ERG11 and changes in its coding sequence, AFR1 encoding drug efflux pumps on chromosome 1, and ABC transporter Pdr11 encoding drug efflux pumps.^[17,18]^ *C gatti* is a genetically and phenotypically distinct species that accounts for 11 to 33% of global cryptococcosis cases.^[19]^ A study by Gomez-Lopez et al reported that the minimum inhibitory concentration (MIC) of azole antifungal drugs against *C gatti* is higher than that against Cryptococcus neoformans, and *C gatti* is more sensitive to amphotericin B and flucytosine.^[20]^ Posaconazole also showed good activity. Beardsley et al reviewed several clinical trials and summarized the average MIC of *C gatti*: fluconazole 1.46 to 8.6 µg/mL, voriconazole 0.02 to 0.10 µg/mL, posaconazole 0.04 to 0.36 µg/mL, and amphotericin B 0.2726 to 0.39 µg/mL.^[19]^ The Clinical and Laboratory Standards Institute (USA) does not have a relevant breakpoint standard for Cryptococcus neoformans and refers to the breakpoints of Candida albicans: fluconazole ≥ 8 to 64 mg/L, itraconazole and voriconazole ≥ 1 mg/L, and amphotericin B ≥ 2 mg/L are considered resistant.^[21]^ Therefore, drug resistance of *C gatti* is still rare. The resistance to fluconazole in case 2 may have been induced by antifungal treatment. Therefore, it is necessary to monitor the MIC of fluconazole before and during treatment when the therapeutic effect is not good. If the MIC exceeds 16, there is a high possibility that the treatment will fail.^[22]^ In case 2, drug susceptibility test showed that Cryptococcus was resistant to fluconazole. That explain why the initial treatment effect of case 2 was poor.

Bacterial persisters: Unlike antibiotic resistance, which reflects the genetic ability of pathogens to grow in the presence of antibiotics above the MIC, tolerance and persistence describe the phenomenon where genetically susceptible pathogen cells survive high doses of bactericides without a change in MIC. These phenomena may be the main reasons for poor therapeutic effects despite clinical drug susceptibility. Bacterial persisters refers to a small subpopulation of pathogenic bacteria that remain in a stationary phase, neither proliferating nor dying, under the pressure of antimicrobial drugs or harsh living conditions. In 2024, the team of Wang Linqi from the Chinese Academy of Sciences achieved nondestructive detection of single cells of Cryptococcus neoformans persisters in the host through Raman spectroscopy.^[23]^ The antioxidant ergothioneine plays a special and critical role in the persistence of amphotericin B. Finally, the authors found that in mice, the antidepressant drug sertraline has a specific killing effect on Cryptococcus persister cells through the strategy of repurposing existing drugs. We look forward to the progress of relevant clinical trials.

Hormone therapy: *C gatti* is more likely to cause encephalitis and meningitis. For example, Case 1 experienced transient ischemic attack (TIA) symptoms during treatment, suggesting that *C gatti* may have caused secondary cerebrovascular inflammatory changes. This raises questions about the benefits of hormone therapy and the risks of infection aggravation. *C gatti* infection mainly involves the lungs and central nervous system. After inhaling yeast into lung tissue, it spreads through blood circulation to enter the central nervous system. It crosses the blood-brain barrier through the cell-to-cell pathway of microvascular endothelial cells, or by hiding in monocytes or using paracellular migration pathways to cross the endothelium (the so-called Trojan horse mechanism), causing intracranial infections.^[24]^ *C gatti* can also distribute to various parts of the brain via blood flow through different blood vessels, causing secondary cerebral vascular inflammatory changes and leading to secondary cerebral infarction.^[25]^ Retrospective analysis has found that hydrocortisone is effective in treating vision loss caused by Cryptococcus meningitis in immunocompromised hosts,^[26]^ and a few cases have shown dexamethasone to be effective in treating intracranial hypertension due to Cryptococcus meningitis.^[27]^ Phillips et al recommended the use of high-dose dexamethasone for patients with severe meningoencephalitis and persistent or worsening symptoms to improve the continuously increasing inflammatory response and reduce intracranial pressure.^[28]^ In patients with *C gatti* infection and extensive lesions in the central nervous system, some cases have described the benefits of glucocorticoid treatment. However, there is insufficient evidence to inform clinicians about the risks or benefits of glucocorticoids for HIV-uninfected patients with cryptococcal meningitis. Recent guidelines recommend against universal use but support short-term treatment for specific indications, such as space-occupying lesions with peripheral mass effects.^[19]^

Detection methods: One of the 2 cases was clearly diagnosed by mNGS, and the other case was identified as *C gatti* infection by mass spectrometry. The clinical testing of Cryptococcus in tertiary hospitals often includes the detection of capsular polysaccharide antigen, cryptococcal ink staining smear, polymerase chain reaction (PCR) detection of cryptococcus DNA, canavanine-glycine-bromothymol blue (CGB) culture medium cultivation, matrix-assisted laser desorption/ionization time-of-flight mass spectrometry (MALDI-TOF MS), histopathological staining, and mNGS. These methods are important for differential diagnosis but have limited utility for evaluating treatment efficacy. Cryptococcus can release capsular polysaccharide antigens continuously after death, leading to a large difference in the accuracy of smear density after centrifugation and enrichment of CSF. Therefore, it is difficult to evaluate treatment efficacy. It is important and meaningful to compare individual patients’ clinical symptoms and signs, biochemical tests of CSF, CSF smears and cultures, specific polysaccharide capsule antigen of Cryptococcus, and MRI of the brain.

This study has several limitations. Firstly, as a case report, its conclusion is based on observations of 2 patients, and *C gatti* infection exhibits significant clinical heterogeneity. Therefore, our findings may not be directly extrapolated to all patient populations. Secondly, as the follow-up period for case 1 has not yet reached 2 years, we are unable to evaluate the long-term efficacy and potential long-term side effects of the treatment plan. Furthermore, although we attempted to analyze our findings through a comprehensive literature review, the review may be limited by database selection and publication bias. Future research needs to collect more cases through multi center collaboration and conduct longer-term follow-up to validate our observations. Further basic research aims to elucidate the pathophysiological mechanisms underlying *C gatti* infection and provide guidance for its more effective treatment.

## 6. Conclusions

Finally, with the advancement of etiological identification technology, *C gatti* infection has been increasingly reported. Its neurological infections are more common than pulmonary infections and often severe in nature. However, due to the relatively low incidence of *C gatti* infections in China, there is a lack of relevant clinical and basic research. This situation requires clinicians to pay attention and improve their ability to identify *C gatti*. We report 2 cases of pulmonary cryptococcosis complicated with cryptococcal encephalitis caused by *C gatti* and it provides new insights for bacterial persisters and hormone therapy. We believe that this report can provide valuable reference for the medical community to identify and manage similar cases. Standardizing the treatment plan for induction, consolidation and maintenance periods, dynamically evaluating efficacy, monitoring fluconazole MIC when necessary, timely surgical intervention, and identifying Cryptococcus neoformans and *C gatti* can help improve therapeutic outcomes. Analyzing these cases may help to elucidate some characteristics of this opportunistic microbial infection. However, due to the small sample size in this study, the findings may not be generalizable to a broader population. Therefore, further research with larger samples is still needed to provide more robust evidence for subsequent diagnosis, treatment plans, and follow-up management.

## Author contributions

**Conceptualization:** Tiegen Xiong, Wei Xiang, Jiake Zhang, Li Li, Hong Xu.

**Data curation:** Yanting Zeng, Tiegen Xiong, Wenyuan He.

**Formal analysis:** Wei Xiang, Wenyuan He.

**Funding acquisition:** Bingmei Deng.

**Investigation:** Wenyuan He.

**Project administration:** Jiake Zhang.

**Software:** Yanting Zeng, Tiegen Xiong, Weifeng Yuan.

**Supervision:** Weifeng Yuan.

**Validation:** Weifeng Yuan.

**Visualization:** Wei Xiang, Bingmei Deng.

**Writing – original draft:** Yanting Zeng, Tiegen Xiong.

**Writing – review & editing:** Li Li, Bingmei Deng, Hong Xu.
